# Developing an Absorption–Based Quality Control Method for Hu–Gan–Kang–Yuan Capsules by UFLC–QTOF–MS/MS Screening and HPLC–DAD Quantitative Determination

**DOI:** 10.3390/molecules21050592

**Published:** 2016-05-18

**Authors:** Fenghuan Wei, Minting Chen, Chaohua Luo, Feilong Chen, Qun Shen, Zhixian Mo

**Affiliations:** College of Traditional Chinese Medicine, Southern Medical University, Guangzhou 510515, China; caddiechen@icloud.com (M.C.); lchua2001@163.com (C.L.); zyxy@smu.edu.cn (F.C.); sq@smu.edu.cn (Q.S.); cherrymo@163.com (Z.M.)

**Keywords:** hu–gan–kang–yuan capsule, quality control, UFLC–QTOF–MS/MS, absorption

## Abstract

Traditional Chinese Medicine Preparations (TCMPs) contain massive numbers of ingredients responsible for their multiple efficacies. An absorption–based quality control method for complicated TCMPs using Hu–gan–kang–yuan Capsule (HGKYC) as an example was developed. To select proper chemical markers for quality control of HGKYC, an ultra–fast liquid chromatography (UFLC) coupled with electrospray ionization quadrupole time–off light mass spectrometry (UFLC–QTOF–MS/MS) method was used for the rapid separation and structural identification of the constituents in the HGKYC extract and the rat serum after oral administration of HGKYC. As a result, one hundred and seven prototype constituents including flavonoids, organic acid, phenylpropanoids, anthraquinones, saponins, alkaloids, terpenes, phenols and amino acids in HGKYC extract, and 43 compounds found in rat serum after oral administration of HGKYC were unambiguously identified or tentatively characterized by comparing retention times and MS information with those of authentic standards or available literature references. Finally, a simple, low–cost and effective method of simultaneous determination for baicalein, wogonin, paeonol and emodin in HGKYC was developed using high performance liquid chromatography coupled with a diode array detector. In conclusion, an absorption–based quality control pattern was developed and successfully used for evaluating HGKYC.

## 1. Introduction

Traditional Chinese Medicine Preparations (TCMPs) are usually composed of multiple Chinese medicinal materials that contain massive numbers of ingredients responsible for their multiple efficacies. In the quality control (QC) of TCMPs, the biggest challenge is the choice of chemical markers due to the complicated nature of the compounds in TCMPs. So far, selecting one compound as the quantitative marker like in Western medicine remains the main QC method used for TCMPs. We investigated and found that among 1493 TCMPs in the Chinese Pharmacopoeia (I volume, 2015 version), 849 TCMPs were evaluated by determining only one marker compound [1]. It is no denying that the Western medicine QC platform is somewhat helpful in evaluating the repeatability of TCMP ingredients among different batches, however, this QC platform may not correlate to the TCMPs’ efficacy, because the compounds selected as quantitative markers may not be active or may not be absorbed. Hence, it is inevitable that sometimes the relationship between the “quality” of a TCMP evaluated by some chemical marker and its pharmacological effects can be inconsistent [2].

Conceptually, the active components which are absorbable should be considered as markers for QC evaluation since they are capable of contributing to the pharmacological activities [3,4], and considering the consistency evaluation among the medicinal materials, semi–finished products and TCMPs, proper prototype compounds absorbed in blood should be the preferred chemical markers. Hence, screening the absorbable compounds in TCMPs is of prime importance before confirming rational chemical markers for QC. The frequently used techniques for screening compounds in TCMPs are liquid chromatography–mass spectrometry (LC–MS) with several mass analyzers, such as ion trap, triple quadrupole (QqQ), quadrupole linear ion trap (QTRAP), time–of–flight (TOF). Q–TOF–MS/MS can analyze the fragment ions with accurate mass measurement, which facilitates the elucidation of characteristic fragmentation pathways of the targeted compounds. Its applications in analyzing the chemical profiles of Traditional Chinese Medicines *in vivo* and *in vitro* are presently hot topic [5,6,7,8,9].

Hu–gan–kan–kang–yuan Capusule (HGKYC) is a second–generation preparation, displaying pharmacological effects in hepatitis B treatment by decreasing aminopherase and increasing immunity. The preparation consists of 12 traditional Chinese medicinal materials, *Schisandrae chinensis fructus*, *Ligustri lucidi fructus*, *Epimedii folium*, *Isatidis Radix*, *Polygoni cuspidate rhizome* et *radix*, *Acanthopanacis senticosi radix* et *rhizomaseu caulis*, *Bupleuri radix*, *Moutan cortex*, *Scutellariae radix*, *Astragali radix*, *Hominis* *placenta*, *Taxilli herba*. Undoubtedly, there are complicated compounds in HGKYC, and which ones can be absorbed in blood should be investigated. This study tries to develop an absorption–based quality control method for TCMPs using HGKYC as an example.

In this paper, firstly an UFLC–QTOF–MS/MS method was used to identify the compounds in HGKYC extract and in rat serum after oral administration of HGKYC. Secondly, based on the pharmacological bioactivities of the absorbable components, the availabilities of authentic standards and the costs of analysis method, baicalein, wogonin, paeonol and emodin were chosen as chemical markers to evaluate the quality of HGKYC by HPLC–DAD. Finally, a simple, reliable and sensitive method for simultaneous determination of the abovementioned compounds was developed.

## 2. Materials and Methods

### 2.1. Chemicals and Materials

The medicinal materials—*Schisandrae chinensis fructus*, *Ligustri lucidi fructus, Epimedii folium*, *Isatidis Radix*, *Polygoni cuspidate rhizome* et *radix*, *Acanthopanacis senticosi radix* et *rhizomaseu caulis*, *Bupleuri radix*, *Moutan cortex*, *Scutellariae radix*, *Astragali radix*, *Hominis placenta*, *Taxilli herba—*were purchased from Kangsheng Medicinal Company (Guanghzou, China). All medicinal materials were identified by associate professor Zhang Hongwei (Department of Medicinal Plants & Pharmacognosy, Southern Medical University, Guangzhou, China) according to pharmacognostic standards documented in Volume I of 2010 Edition of China Pharmacopoeia. All samples were kept in a desiccator (silica gel as desiccant) at room temperature in Department of Chinese Pharmaceutics, Southern Medical University, Guangzhou, China. Three batches of Hu–gan–kang–yuan capsules were prepared in the laboratories of the Department of Chinese Pharmaceutics, Southern Medical University.

The reference standards of baicalein, wogonin, paeonol and emodin, baicalin, isofraxidin, deoxyschizandrin, schisandrin, paeonol, oroxylin A, salidroside, emodin, paeoniflorin, polydatin, scutellarin, wogonin, resveratrol, oleanic acid, syringing, rhein, ligustroflavone, chrysin, baicalein, ferulic acid, epigoitrin, protocatechuate and astragaloside A were purchased from the National Institute for the Control of Pharmaceutical and Biological Products (Beijing, China). Arginine, choline, betaine, adenine, nicotinic acid, l–pyroglutamic acid, tyrosine, isoleucine, p–coumaric acid, gallic acid, phenylalanine, tryptophan, protocatechuic aldehyde, methyl gallate, chlorogenic acid, caffeic acid, syringic acid, *trans*–p–coumaric acid, rutin, scutellarin, hyperoside, quercetrin, ethyl caffeate, kaempferol, schizandrin A and r–schizandrin were purchased from Wei Ke Qi Biological Technology Co., Ltd. (Chengdu, China). The purities of all the standards were greater than 98.0%. Acetonitrile was chromatographic grade, and phosphoric acid, methanol, ethanol were analytical grade. All of them were purchased from Guangzhou Chemical Reagent factory (Guangzhou, China). Ultrapure water was provided by Southern Medical University (Guangzhou, China).

Three male Sprague–Dawley rats weighing 260 ± 20 g were obtained from the Animal Center of Southern Medical University. Animals were bred in a breeding room with a temperature of 23 ± 2 °C humidity of 60% ± 5%, and 12 h dark–light cycle. They were given tap water and fed a normal diet and were acclimatized to the facilities for 3 days. The rats were fasted for 12 h before experimentation, while water was taken freely. The animal experiments were carried out in accordance with the Guide for the Care and Use of Laboratory Animals of Southern Medical University.

### 2.2. Instrumentation and Conditions

#### 2.2.1. UFLC–QTOF 5600^+^MS/MS

UFLC analysis was performed on a Shimadzu UFLC XR instrument (Shimadzu Corp., Kyoto, Japan), consisting of a binary pump, an auto–sampler, a column oven and a diode–array detector. Samples were separated on a Kinetex C_18_ column (100 mm × 2.1 mm I.D., 2.6 μm, Phenomenex, CA, USA). The mobile phase consisted of acetonitrile (A) and 0.1% aqueous formic acid (*v*/*v*) (B). The following gradient elution program was used—linear gradient from 2% to 100% A at 0–30 min), isocratic 100% A at 30–40 min), 100%–2% A at 40–41 min), isocratic 2% A at 41–45 min. The flow rate was kept at 0.3 mL/min. The injected volume was 2 μL and the column temperature was set at 40 °C. The DAD detector scanned from 190 nm to 400 nm. Mass spectrometry was performed on the Triple TOF^TM^ 5600 plus (AB SCIEX, Foster City, CA, USA) a hybrid triple quadrupole time–of–flight mass spectrometer equipped with ESI source. The system was operated with Analyst^®^TF 1.6 software (AB SCIEX). The conditions of the MS/MS detector were as follows: first ion source gas 60 psi, second ion source gas 60 psi, curtain gas 35 psi, temperature 550 °C, ion spray voltage floating 5500 V, collision energy 35 V, collision energy spread 15 V, declustering potential 80 V. TOF–MS range was set at *m/z* 100–1000 and product ions mass range was set at *m/z* 50–1000. Both positive and negative ion modes were used for compound ionization. Nitrogen was used as nebulizer and auxiliary gas.

#### 2.2.2. HPLC–DAD 

Quantitative analysis of baicalein, wogonin, paeonol and emodin was carried out in a 1260 HPLC system (Agilent Technologies, Palo Alto, CA, USA) equipped with a diode–array detector The separation was achieved on a Venusil XBL C_18_ (250 mm × 4.6 mm I.D., 5 µm) (Dalian Elite Analytical Instruments Co., Ltd., Dalian, China) at ambient temperature. The mobile phase consisted of acetonitrile (solvent A) and 0.1% phosphoric acid in water (solvent B). The gradient used is as follows: 30% A at 0–4 min, 30%–25% A at 4–5 min, 25% A at 5–10 min, 25%–40% A at 10–25 min, 40%–70% at 25–32 min, 70%–76% at 32–35 min, 76%–85% at 35–45 min, 85%–30% at 45–50 min. The flow rate was maintained at 1 mL/min, the injection volume was 10 µL and the eluent was monitored at 280 nm.

### 2.3. Animal Administration

Three SD rats were orally administrated HGKYC powder 10 g/kg. Blood was collected from caudal vein at 4 h after administration and centrifuged at 6000 *g* for 10 min at 4 °C. Serum was frozen at −20 °C until analysis.

### 2.4. Sample Preparation

#### 2.4.1. Extract of HGKYC

The contents of HGKYC (0.20 g) were weighed precisely and treated by sonication with 10 mL of methanol for 15 min at 50 °C in an ultrasonic bath (JY92–II, frequency 40 kHz, 250 W, Ningbo Biotechnology Co., Ltd., Ningbo, China). The extract solution was centrifuged at 13,000 rpm for 15 min. Then the supernatant was injected into UFLC and HPLC.

#### 2.4.2. Rat Serum Sample

For identification of multiple constituents in rat, the serum was vortex mixed with 2.5 times amount of acetonitrile (*v*/*v*) to precipitate proteins and centrifuged at 13,000 *g* for 15 min. The supernatant was injected into the UFLC–Q–TOF MS/MS system.

#### 2.4.3. Preparation of Standard Solutions for Qualitative Identification

Known amounts of reference standards arginine, choline, betaine, adenine, nicotinic acid, l–pyroglutamic acid, tyrosine, isoleucine, p–coumaric acid, gallic acid, phenylalanine, tryptophan, protocatechuic aldehyde, methyl gallate, chlorogenic acid, caffeic acid, syringic acid, *trans–*p–coumaric acid, rutin, scutellarin, hyperoside, quercetrin, ethyl caffeate, kaempferol, schizandrin A, r–schizandrin B, baicalin, isofraxidin, deoxyschizandrin, schisandrin, paeonol, oroxylin A, salidroside, emodin, paeoniflorin, polydatin, scutellarin, wogonin, resveratrol, oleanic acid, syringing, rhein, ligustroflavone, chrysin, baicalein, ferulic acid, epigoitrin, protocatechuate and astragalosfide A were dissolved in methnaol to obtain single stock solultions (about 1.0 mg/mL) and stored at 4 °C until used.

The working solutions were prepared by serial dilution of the stock solutions with methanol. A mixture of all forty–nine reference standards was prepared in methanol and was filtered through a 0.22 μm syringe filter before UFLC–QTOF–MS/MS analysis.

#### 2.4.4. Preparation of Standard Solutions for Quantitative Determination

Standard stock solutions of bacalein, wogonin, paeonol and emodin were precisely weighed and prepared by dissolving them in methanol, respectively. A mixed standard solution was prepared by accurately transferring each–standard stock solution and diluting with methanol. The final concentration of baicalein, wogonin, paeonol and emodin in the mixed standard working solution was 40.25, 46.75, 32.12 and 26.70 μg/mL, respectively. The quality control samples (LQC, MQC and HQC) were 5, 50, and 70 μg/mL for bacalein, 5, 50 and 80 μg/mL for wogonin, 4, 20 and 40 μg/mL for paeonol and emodin, respectively. All solutions were stored at 4 °C until used.

#### 2.4.5. Extract of Negative Control Sample Solution

The negative control samples of HGKYC were prepared by getting rid of one corresponding material medicine from this preparation. The herbs were ground into powders before use, and prepared using the sample preparation protocol (showed in Table 1).

### 2.5. Establishment of Peak Identification

The UFLC–Q–TOF–MS/MS data of samples were analyzed by PeakView software version 1.2 (AB SCIEX), mainly with the XIC manager tool which provided the quasi–molecular weight, mass errors and isotope pattern fit. When a reference standard was available, the compound was identified by comparing the retention time and MS/MS spectra. While the identification of compound without available reference standard was mainly based on MS/MS data and available literature reports. The mass error of molecular ions of all identified compounds was less than ±5 ppm.

### 2.6. Method Validation for Determination of Baicalein, Wogonin, Paeonol and Emodin in HGKYC

A HPLC–DAD method was developed for simultaneous determination of baicalein, wogonin, paeonol and emodin for HGKYC quality evaluation. Specificities were assessed by comparing chromatograms of a standard solution mixture of baicalein, paeonol, wogonin and emodin, extract solution of HGKYC and negative control samples under the same conditions. Linearities were assessed by assaying calibration curves with peak area *vs.* five different injection amounts of 0.040–0.805 μg, 0.032–0.482 μg, 0.047–0.935 μg and 0.027–0.534 μg for baicalein, paeonol, wogonin and emodin, respectively. The intra–day and inter–day precisions (%, RSD) were established by analyzing QC samples on day 1 and on each of three consecutive days in five replicates. The accuracies were evaluated by means of recovery assays performed by adding known amounts of baicalein, paeonol, wogonin and emodin standards to the sample at the similar concentration in six replicates. The spiked amounts of baicalein, paeonol, wogonin and emodin were 77.10, 96.60, 26.70 and 28.05 μg, respectively. The original amount of baicalein, paeonol, wogonin and emodin in sample solution was 85.23, 104.12, 28.11 and 30.34 μg, respectively. The recoveries were calculated as follows:

Recovery (%) = [(determined amount–original amount)/amount spiked] × 100
(1)

The stabilities were evaluated by RSD (%) of peak area of baicalein, paeonol, wogonin and emodin by analyzing sample solution in six replicates at 4.0 °C within 1 month.

## 3. Results

### 3.1. Optimization of UFLC–QTOF–MS/MS Conditions

The ingredients of HGKYC belonged to different chemical families and showed distinct polarities. Thus, to obtain an effective separation, guarantee high ionization, and minimize ion suppression, the mobile phase system consisting of a mixture of 0.1% formic acid water solution and acetonitrile with gradient elution was used. The MS conditions, such as the parameters of gas pressure, ion spray voltage, capillary temperature and voltage of declustering potential were optimized. The optimized conditions were described in Section 2.2.1*.* The positive and negative ion modes were detected.

### 3.2. Profiles of Ingredients from HGKYC Extract

**Figure 1:**
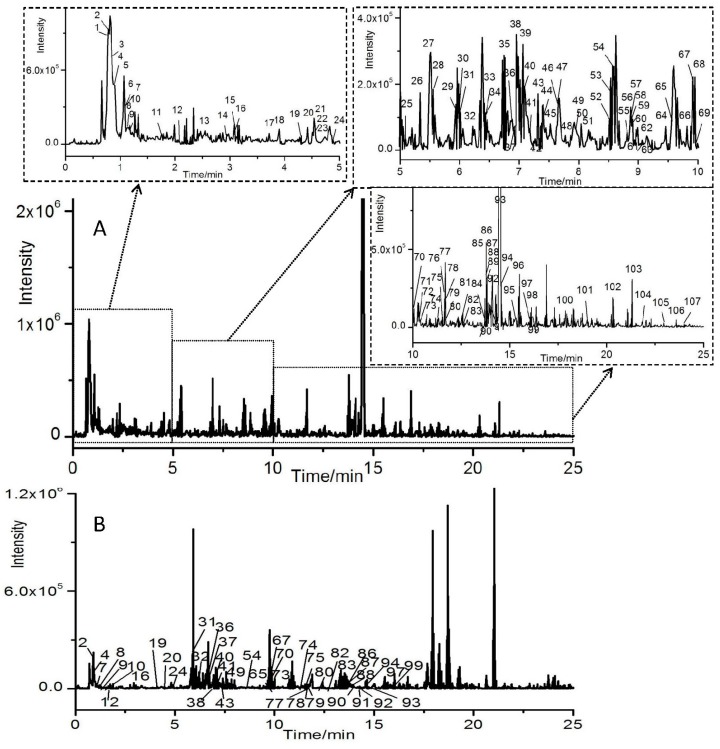
Total ion chromatography of HGKYC extract (**A**) and rat serum after oral administration of HGKYC (**B**) in positive ion mode by UFLC–QTOF–MS/MS. The enlarged view of the area marked was shown on the top and top right.

The total ion chromatograms (TIC) of HGKYC in positive and negative ion modes were obtained. Figure 1A showed the representative positive signal of samples. To better understand the MS fragmentation patterns of the constituents, forty nine of reference standards, including flavonoids, isoflavonoids, saponins, anthraquinones, organic acid and iridoids were investigated by UFLC–Triple–QTOF–MS/MS firstly.

Under the present conditions, a total of 107 compounds from HGKYC were identified, forty nine of which were unambiguously identified by comparing their retention times, accurate masses and fragment ions with those of the available reference standards, while the others were tentatively identified by elucidating the quasi–molecular ions, fragment ions as well as the available literature reports. The data information of compounds are summarized in Table 2, which includes retention time, molecular formula, measured mass, fragment ions, compound name and related literatures.

#### 3.2.1. Identification of Flavonoids and Isoflavonoids

There are notable amounts of flavonoids and flavonoid derivatives in *Scutellariae radix* and *Epimedii folium*. Especially there are amounts of prenylated flavones in *Epimedii folium*, which have characteristic prenylated group at C–8 position. In MS analysis, the consecutive losses of sugar, H_2_O, carbonyl and isopentene group (C_4_H_8_) were observed as typical fragmentation profiles of these prenylated flavones. A total of 45 flavones or isoflavones were identified in HGKYC, with 18 definitely elucidated and the others tentatively identified.

The fragmentation patterns of ordinary flavones obey the Retro–Diels–Alder (RDA) fragmentation mechanism and the losses of a glucose residue (162 Da), rhamnose residue (146 Da), a methyl group (15 Da), H_2_O (18 Da), CO (28 Da) which are often the initial breakdown syeps of flavonoids [32]. For instance, Peak 54 gave an [M + H]^+^ ion at *m/z* 447.0912 (C_21_H_18_O_11_) and yielded a high intensity fragment ion at *m/z* 271.0595 by loss of one glucuronic acid moiety (176 Da), and peak 79 gave an [M + H]^+^ ion at *m/z* 271.0596 and a fragment ion at *m/z* 123.0080 by loss of H_2_O (18 Da). It indicated that peak 54 might be the glucuronide of peak 79, by comparing retention time, UV spectra and the fragment ions with the reference standards, so peaks 54 and 79 were identified as baicalin and baicalein, respectively [23]. Peak 81 gave an [M + H]^+^ ion at *m/z* 269.0805 (C_16_H_12_O_4_) in positive ion mode, and by comparing the retention time and fragmentation pathways with the authentic standard, peak 81was identified as formononetin. Peak 56 gave an [M + H]^+^ ion at *m/z* 431.1318 (C_22_H_22_O_9_) and yielded a characteristic fragment ion at *m/z* 269.0802 by losing one glucose residue, which indicated peak 56 possibly was the glycoside of peak 81. By comparing MS/MS data with peak 81 and the available literature, peak 56 was tentatively identified as formononetinglucoside (ononin) [10]. Peaks 39, 40, 41, 46, 54, 78, 69, 70, 75, 77, 81, 86, 88, 91 were definitely identified as rutin, scutellarin, hyperoside, ligustroflavone, baicalin, baicalein, wogonoside, oroxyloside, kaempferol, isorhamnetin, formononetin, wogonin, oroxylin A and chrysin, respectively, by comparing their MS and UV spectra information with those of reference standards.

The loss of an isopentene group was observed as a typical fragmentation profile of prenylated flavones except the consecutive neutral losses of sugar moieties. For example, Peak 72 gave an [M + H]^+^ ion at *m/z* 531.1846 and yielded characteristic fragment ions at *m/z* 369.1326 and 313.0695 by successive losses of glucose residue (162 Da) and isopentene group (56 Da). By comparing with the information of authentic standard, peak 72 was identified as icariside I [14]. Peak 73 gave an [M + H]^+^ ion at *m/z* 677.2427 and gave fragment ions at *m/z* 531.184, 369.1332 and 313.0712 by the successive losses of one rhamnose, glucose residue and isopentene group, peak 73 was identified as icariin by comparing with the authentic standard [14]. Based on the characteristic fragmentation profiles of flavones and prenylated flavones as well as the available literatures, 27 flavones (peaks 38, 43, 44, 48, 49, 60, 64, 65, 66, 67, 76, 51, 52, 53, 59, 61, 62, 71, 78, 80, 83, 87, 89, 90, 92, 94 and 98) were tentatively identified (Table 2).

Peak 51 gave an [M + H]^+^ ion at *m/z* 679.2215 (C_32_H_38_O_16_) in positive ion mode and yielded fragment ions at *m/z* 533.1669, 371.1096 and 315.0457 by successive losses of one rhmnose residue, glucose residue and isopentene group, it was tentatively identified as hexandraside E by comparison with the available literature [14]. Peak 53 gave an [M + H]^+^ ion at *m/z* 517.1693 (C_26_H_28_O_11_) in positive ion mode and yielded a high intensity fragment at *m/z* 355.1191 by loss of one glucose, while another fragment ion at *m/z* 299.0524 originated from the ion at *m/z* 355.1191 by losing one isopentene group. Additionally, peak 53 had the same fragment patterns as diphylloside B after the latter lost the two–connected rhamnoses, so by comparison with the available literature, peak 53 was speculated to be icarrin C [14]. Peak 61 gave an [M + H]^+^ ion at *m/z* 839.2967 (C_39_H_50_O_20_) in positive ion mode and yielded characteristic fragment ions at *m/z* 677.2346, 531.1881, 369.1342 and 313.0691 by the successive losses of one glucose, rhamnose, C–7–O–glucose and isopentene group, and by comparison with the available literature, peak 61 was tentatively identified as epimedin A [14]. Peak 62 gave an [M + H]^+^ ion at *m/z* 693.2365 (C_33_H_40_O_16_) in positive ion mode and yielded fragment ions at *m/z* 547.1791 and 531.1895 by the losses of one rhamnose and glucose residue, respectively. The fragment ion at *m/z* 385.1284 was originated by the simultaneous losses of one rhamnose and glucoseresidue from the molecular ion. The characteristic fragment ion at *m/z* 313.0687 originated from the fragment ion at *m/z* 369.1321 by the loss of an isopentene group. By comparison with the available literature, peak 62 was tentatively identified as anhydroicaritin–3,7–di–O–glucoside [14].

#### 3.2.2. Identification of Organic Acids

Because of the presence of COOH groups in organic acid structures, their fragment ions were usually generated by the losses of H_2_O (18 Da), CO_2_ (44 Da) and HCOOH (44 Da). Seventeen organic acids have been identified from HGKYC, nine of which were confirmed by reference standards, peaks 6, 10, 11, 22, 24, 31, 32, 35 and 106 were identified as nicotinic acid, p*–*coumaric acid, gallic acid, chlorogenic acid, caffeic acid, syringic acid, *trans–*p–coumaric acid, ferulic acid and oleanic acid, respectively. The other organic acids (peaks 13, 14, 20, 29, 45, 97, 105, 107) were tentatively assigned. Peak 14 exhibited an [M − H]^−^ ion at *m/z* 153.0199 and yielded high intensity fragments ions at *m/z* 109.0309 and 91.0208 by the successive losses of CO_2_ and H_2_O, by comparing with the literature report, peak 14 was identified as protocatechuic acid [13]. Peak 20 gave an [M − H]^−^ ion at *m/z* 341.0881 (C_15_H_18_O_9_) and yielded fragment ions at *m/z* 179.0352 and 135.0454 by the successive losses of one glucose residue and CO_2_, so was inferred that peak 20 was caffeoylglucose by comparison with the available literature [13]. Peak 97 gave an [M + H]^+^ ion at *m/z* 489.3566 (C_30_H_48_O_5_) in positive ion mode and yielded major fragment ions at *m/z* 453.3324 and 407.3254 by the successive losses of 2H_2_O and HCOOH, respectively. By comparison with the available literature, peak 97 was tentatively identified as tormentic acid [15]. Peak 107 had the same [M − H]^−^ ion at *m/z* 455.3537 (C_30_H_48_O_3_) with oleanic acid in negative ion mode. Oleanic acid and ursolic acid were reported in *Ligustri lucidi fructus*, therefore, peak 107 was tentatively characterized as ursolic acid [15]

#### 3.2.3. Identification of Phenylpropanoids

Fifteen phenylpropanoids have been identified from HGKYC, seven of which were identified by comparison with the available reference standards. Peaks 23, 25, 33, 37, 93, 102 and 103 were identified as syringoside, isofraxidin, eleutheroside E, resveratrol, schizandrin A and γ–schizandrin B by comparing with their reference standards, respectively. Peak 96 gave an [M + H]^+^ ion at *m/z* 417.1890 (C_23_H_28_O_7_) in positive ion mode and yielded fragment ions at *m/z* 399.1815 and 384.1501 by the successive losses of H_2_O, CH_3_ or OCH_3_. The [M + H − H − 18 − 42]^+^ ion at *m/z* 357.1394 originated from the ion at *m/z* 417.1890 by loss of H_2_O and C_3_H_6_. By comparison with the available literature, peak 96 was tentatively identified as schisandrol B [31]. Peak 100 gave an [M + H]^+^ ion at *m/z* 403.2112 (C_23_H_30_O_6_) in positive ion mode and yielded fragment ions at *m/z* 388.1848, 372.1919 and 371.1859 by the losses of CH_3_, OCH_3_ and CH_3_OH, respectively. Peak 100 was tentatively identified as schisanhenol based on the available literature [31]. Peak 101 gave an [M + H]^+^ ion at *m/z* 515.32264 (C_28_H_34_O_9_) in positive mode and yielded characteristic fragment ion at *m/z* 415.2009 by the loss of C_4_H_6_COOH. The ion at *m/z* 385.1637 was originated from the ion at *m/z* 415 by the loss of one formaldehyde. By comparison with the available literature, peak 101 was tentatively identified as schisantherin B [31]. Peak 104 gave an [M + H]^+^ ion at *m/z* 385.1638 in positive mode and yielded fragment ions at *m/z* 355.1561 and 315.0895 by the loss of OCH_2_ and C_5_H_10_. The ion at *m/z* 285.0757 was originated from ion at *m/z* 315.0895 by the loss of OCH_2_. By comparison with the available literature [31], peak 104 was identified as schizandrin C.

#### 3.2.4. Identification of Anthraquinones

Three anthraquinones have been identified from HKGYC, one of which was identified by comparing with the authentic standard, namely peak 99 identified as emodin [18]. Peaks 27 and 57 were tentatively assigned as mangiferin and polygonimitin B based on the available literature reports [18].

#### 3.2.5. Identification of Saponins

Three saponins have been identified from HKGYC. Peak 95 gave an [M + H]^+^ ion at *m/z* 869.4881 and yielded fragment ions at *m/z* 689.4234, 437.3396, 217.0720 and 157.0506. By comparison with an authentic standard, peak 95 was identified as astragaloside A [10]. Peak 84 gave an [M + H]^+^ ion at *m/z* 827.4768 (C_43_H_70_O_15_) in positive ion mode and gave a series of fragment ions typical of triterpenoid saponins at *m/z* 455,437,175 and 143; peak 85 gave an [M + H]^+^ ion at *m/z* 781.4736 (C_42_H_68_O_13_) and yielded fragment ions at *m/z* 745.4447,619.4203, 583.3921, by comparison with the available literature, peaks 84 and 85 were tentatively identified as astragaloside II and saikosaponin a, respectively [18,23].

#### 3.2.6. Identification of Alkaloids

Four alkaloids were identified from HGKYC, of which peaks 2, 4 and 15 were definitely identified as choline, betaine and epigoitrin by comparison with their authentic standards [18,19]. Peak 26 gave an [M + H]^+^ ion at *m/z* 342.1695 (C_20_H_23_O_4_N) and gave an [M − H]^−^ ion at *m/z* 340.1550 in negative mode, and yielded [M + H − H − HCN − H_2_O]^+^ at *m/z* 297.1110 by losing HCN and H_2_O, so peak 26 was tentatively identified as magnoline on the basis of the available literature [14].

#### 3.2.7. Identification of Terpenes and Phenols

Peaks 17, 18, 19, 21, 30, 36, 68, 74 were identified as protocatechuic aldehyde, salidroside, methyl gallate, catechin, paeoniflorin, polydatin, caffeate and paeonol by comparison with authentic standards. Peak 63 gave an [M − H]^−^ ion at *m/z* 599.1787 (C_30_H_32_O_13_) and yielded fragment ions at *m/z* 581.1699, 477.1593, 449.7983 by the losses of H_2_O, benzoyloxy and CO. The [M − H − 122 − 28]^−^ ion at *m/z* 449.7983 originated from the ion at *m/z* 599 by losing benzoyloxy and CO, which indicated the presence of a benzoyloxy group. Based on the fragmentation pathways of peak 30 and literature data [20], peak 63 was tentatively identified as benzoyloxypaeoniflorin. Peak 47 gave an [M − H]^−^ ion at *m/z* 685.2372 in negative ion mode and had fragment ions at *m/z* 685.2439, 523.1866, 453.1445 and 421.1530. Peak 47 was tentatively identified as specnuezhenide based on the published literature [15].

#### 3.2.8. Identification of Other Compounds

By comparing the retention times and MS spectra with those of authentic standards, peaks 1, 5, 7, 8, 9, 12 and 16 were identified as arginine, adenine, l–pyroglutamic acid, tyrosine, isoleucine, phenylalanine and tryptophan, respectively. Peak 3 gave an [M + H]^+^ ion at *m/z* 116.0706 (C_5_H_9_NO_2_) in positive ion mode and yielded a characteristic ion at *m/z* 70.0671 by the loss of HCOOH; peak 28 gave an [M − H]^−^ ion at *m/z* 377.1450 (C_17_H_20_N_4_O_6_) and yielded fragment ions at *m/z* 359.1339 and 243.0878 by the losses of H_2_O and C_5_H_10_O_4_ group, because of the unavailability of authentic standards, peak 3 and peak 28 were tentatively identified as proline and riboflavin, respectively, based on the available literature [32,33].

### 3.3. Profiles of Ingredients in Rat Serum after Oral Administration HGKYC

By comparing retention times, UV spectra and MS/MS data with those of compounds analyzed in HGKYC extract sample, forty three prototype compounds were found in rat serum (shown in Table 2 and Figure 1B). As shown in Table 2, the compounds absorbed in blood were far fewer than those in HGKYC extract, which indicated the number of compounds absorbed in blood is limited due to poor absorption or low concentration. On the basis of the availabilities of authentic standards and pharmacological effects reported, baicalein, wogonin, paeonol and emodin were then chosen as the chemical markers for quality control of HGKYC.

### 3.4. Optimization of HPLC Conditions

**Figure 2:**
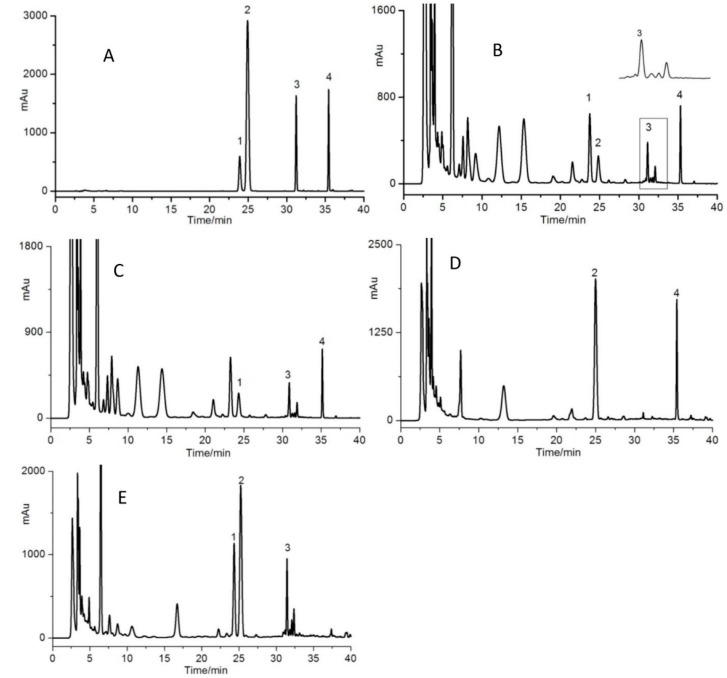
HPLC chromatographic profiles of standards mixture (**A**); sample of Hu–gan–kang–yuan capsule (**B**); negative control sample without *Moutan cortex* (**C**); without *Scutellariae radix* (**D**); and without *Polygoni cuspidate rhizome et radix* (**E**). 1: baicalein, 2: paeonol, 3: wogonin, 4: emodin.

In this study, different mobile phases, elution modes and detection wavelengths were investigated. Mobile phases of acetonitrile–water and methanol–water with different modifiers including acetic acid, formic acid and phosphoric acid were tested under different gradient elution modes. The detection wavelength was selected according to the maximum absorption wavelengths of baicalein, wogonin, paeonol and emodin shown in UV spectra from DAD. The optimized conditions were described in Section 2.2.2. Finally, excellent separations were achieved and typical chromatograms are shown in Figure 2.

### 3.5. Optimization of Extraction Conditions

Ultrasound–assisted extraction (UAE) has been widely used in sample preparation for the quality control of TCMPs. In the extraction process, extraction solvent, sample–solvent ratio, extraction time and temperature are critical for high extraction effectiveness. Pure and aqueous methanol or ethanol solutions are often used as extraction solvents. In the present study, based on the physicochemical properties of the targeted compounds, different concentrations (50%, 80%, and 100%) of methanol water solutions were examined to extract the four compounds in HGKYC. Considering extract rates of targeted compounds, contents of interfering components and extract time, samples were extracted for 15 min by UAE using 50–time of methanol, 50 °C as extraction temperature.

### 3.6. Method Validation

Typical HPLC chromatograms of the authentic standards of the four compounds, the sample, and the negative control samples were shown in Figure 2. This showed that there are no peaks of baicalein, paeonol, wogonin and emodin in the corresponding negative control samples. In addition, targeted compounds in the reference standard sample and the tested sample showed good resolution with the adjacent peaks. The calibration curves, coefficients of determination (r) and concentration ranges were y = 27858x–115.36 (r = 1.0) for baicalein in 0.040–0.805 μg, y = 32233x + 38.381 (r = 0.9998) for paeonol in 0.032–0.482 μg, y = 30503 x + 96.12 (r = 1.0) for wogonin in 0.047–0.935 μg and y = 46970 x + 321.12 (r = 0.9994) for emodin in 0.027–0.534 μg, respectively. The mean recoveries of baicalein, paeonol, wogonin and emodin were 98.45% (RSD = 2.53%), 97.55% (RSD = 2.84%), 99.13% (RSD = 1.54%) and 101.08% (RSD = 2.04%), respectively. The intra–day precisions (RSD values) of baicalein, paeonol, wogonin and emodin were in the range of 0.33%–1.82%, 1.21%–1.97%, 0.63%–1.52% and 0.73%–1.92%, respectively, the inter–day precisions were in the range of 1.16%–1.65%, 1.52%–1.95%, 1.33%–1.59% and 1.13%–1.79%, respectively. The RSD values of baicalein, paeonol, wogonin and emodin solution at 4.0 °C within 1 month were in the range of 1.1%–2.17%, 1.58%–2.95%, 1.22%–2.15% and 0.98%–2.99%, respectively.

### 3.7. Sample Determination

The contents of baicalein, paeonol, wogonin and emodin in HGKYC were determined using the validated methods in triplicates. The results are shown in Table 3.

## 4. Discussion

UFLC–QTOF–MS/MS, a high–efficiency analytical technique, can provide qualitative and quantitative information about samples, but the expensive instrument price and analysis cost limits its applicability in quality control of TCMPs for pharmaceutical companies. HPLC–DAD is a high quality and inexpensive analysis technique, commonly used to analyze quantitatively chemical markers in TCMPs. At present, HPLC–DAD is irreplaceable in QC of TCMPs though it is not as powerful as UFLC–QTOF–MS/MS in functional analysis. Ideally, the more chemical markers selected to evaluate the quality of TCMPs, the better to ensure the consistency between the quality of TCMPs and their pharmacological activities, but that is unpractical because of the limited availability of authentic standards. In this paper, on the basis of the prototype compounds detected in rat serum after oral administration HGKYC, considering the following reasons: (1) the analysis cost; (2) the popularity of analysis equipment; (3) the availabilities of authentic standards; and (4) the reported anti–inflammatory activities and protective effects on hepatocytes of baicalein and wogonin in *Scutellariae radix* [34,35,36,37], paeonol in *Moutan Cortex* [38,39] and emodin in *Polygoni Cuspidate Rhizome* et *Radix* [40,41], then baicalein, wogonin, emodin and paeonol were selected as chemical markers for evaluating the quality of HGKYC by HPLC–DAD. Hence, the quality control scheme for HGKYC using the absorption–based chemical markers is warranted.

## 5. Conclusions

In summary, a reliable and powerful twenty–five minute long analytical method by UFLC–Q–TOF–MS/MS was successfully established for identifying compound profiles in HGKYC extract and in rat serum after oral administration of HGKYC. A total of 107 compounds in HGKYC, 43 compounds of which were also present in rat serum, were identified or tentatively identified based on their retention times, UV spectra, and MS information. The absorption–based quality control scheme for HGKYC provides a valuable demonstration for the quality control of TCMPs.

## Figures and Tables

**Table 1:** Information and preparation protocols of negative control samples.

NC Samples	Preparation Method	Specificity Evaluation
Without *Scutellariae radix*	①	baicalein and wogonin
Without *Moutan cortex*	②	paeonol
Without *Polygoni cuspidate rhizome et radix*	③	emodin

① All medicinal materials in Hu–gan–kang–yuan capsule, except for *Scutellariae radix*, were exactly weighed and exacted according to the Section 2.4.1. The extract solution was used as negative control sample to evaluate specificity for determining baicalein and wogonin; ② All medicinal materials in Hu–gan–kang–yuan capsule, except for *Moutan cortex*, were exactly weighed and exacted according to the Section 2.4.2. The extract solution was used as negative control sample to evaluate specificity for determining paeonol; ③ All medicinal materials in Hu–gan–kang–yuan capsule, except for *Polygoni cuspidate rhizome* et *radix*, were exactly weighed and exacted according to the Section 2.4.3. The extract solution was used as negative control sample to evaluate specificity for determining emodin.

**Table 2:** Identified compounds in extract of HGKYC and in rat serum after oral administration of HGKYC in positive and negative ion mode.

No.	T_R_ (min)	Formula	[M + H]^+^ (Error, ppm)	[M − H]^−^ (Error, ppm)	Fragment Ions in		Identified Compounds		Ref.
					Positive (+) Ion Mode	Negative (−) Ion Mode	In Extract	In Serum	
1	0.77	C_6_H_14_N_4_O_2_		173.1046 (1.2)		131.0829	Arginine ^s^	−	[10]
2	0.78	C_5_H_13_NO	104.1070 (0.7)		60.0832 [M + H − H − C_2_H_5_O]^+^		Choline ^s^	+	[10]
					58.0680				
3	0.85	C_5_H_9_NO_2_	116.0705 (−0.7)		70.0671 [M + H − HCOOH]^+^		Proline	−	[11]
4	0.9	C_5_H_11_NO_2_	118.0861 (−0.7)	116.0721 (3.5)	72.0818 [M + H − HCOOH]^+^		Betaine ^s^	+	[10]
					58.0676, 55.0566				
5	1.07	C_5_H_5_N_5_	136.0717 (−0.2)		119.0353 [M + H − H − NH_3_]^+^		Adenine ^s^	−	[10]
					92.0251 [M + H − H − NH_3_ − HCNN]^+^				
6	1.08	C_6_H_5_NO_2_	124.0392 (−0.5)		106.0278 [M + H − H_2_O]^+^		Nicotinic acid ^s^	−	
					79.0425, 78.0348				
7	1.08	C_5_H_7_NO_3_	130.0499 (−0.6)		84.0461 [M + H − HCOOH]^+^		l-Pyroglutamic acid ^s^	+	[10]
					56.0530 [M + H − H − NH_3_ − HCNOOH]^+^			+	
8	1.13	C_9_H_11_NO_3_	182.0806 (−3.3)		165.0547 [M + H − H − NH_3_]^+^		Tyrosine ^s^	+	[10]
					136.0761 [M + H − HCOOH]^+^				
					119.0487 [M + H − HCOOH − H_2_O]^+^				
					91.0548				
9	1.17	C_6_H_13_NO_2_	132.1018 (−1.1)		86.0964 [M + H − HCOOH]^+^,		Isoleucine ^s^	+	[10]
					69.0711 [M + H − HCOOH − NH_3_]^+^				
10	1.21	C_9_H_8_O_3_	165.0546 (0.02)		123.0448 [M + H − H − C_2_H_2_O]^+^		p-Coumaric acid ^s^	+	[10]
					95.0500 [M + H − H − C_2_H_2_O − CO]^+^				
					77.0397 [M + H − H − C_2_H_2_O − CO − H_2_O]^+^				
					65.0414				
11	1.73	C_7_H_6_O_5_	171.0285 (−1.7)	169.0143 (0.4)	153.0180 [M + H − H_2_O]^+^	125.0253 [M − H − CO_2_]^−^	Gallic acid ^s^	−	[10]
					107.0131 [M + H − H_2_O − HCOOH]^+^	79.0217 [M − H − CO_2_ − HCOOH]^−^			
12	2.04	C_9_H_11_NO_2_		164.0716 (−0.9)		147.0438 [M − H − NH_3_]^−^	Phenylalanine ^s^	+	[10]
						103.0560 [M − H − NH_3_ − CO_2_]^−^			
13	2.54	C_8_H_8_O_4_		167.0350 (0.2)		123.0449 [M − H − CO_2_]−	Vanillic acid	−	[12]
						93.0358 [M − H − CO_2_ − OCH_3_]^−^			
						97.0283, 69.0341, 65.0399			
14	2.92	C_7_H_6_O_4_		153.01989 (3.6)		109.0309 [M − H − CO_2_]^−^	Protocatechuic acid	−	[13]
						91.0208 [M − H − CO_2_ − H_2_O]^−^			
15	3	C_5_H_7_NOS	130.0316 (−3.9)	128.0442 (1.0)	103.0536 [M + H − HCN]^+^		Epigoitrin ^s^	−	[14]
					84.0819 [M + H − HCOOH]^+^				
					70.0657, 60.9750				
16	3.08	C_11_H_12_N_2_O_2_		203.0825 (−0.4)		142.0664 [M − H − NH_3_ − CO_2_]^−^	Tryptophan ^s^	+	[10]
						116.0513 [M − H − NH_3_ − 2OCH_2_]^−^			
17	3.83	C_7_H_6_O_3_		137.0248(2.8)		119.0145 [M − H− H_2_O]^−^	Protocatechuic aldehyde ^s^	−	[13]
						108.0288 [M − H − CO]^−^			
						92.0284			
18	3.87	C_14_H_20_O_7_		299.1140 (1.3)		137.0244 [M − H − Glc]^−^	Salidroside ^s^	−	[15]
						119.0335 [M − H − Glc − H_2_O]^−^			
						93.0357, 59.0172			
19	4.31	C_8_H_8_O_5_		183.0300 (0.7)		168.0065 [M − H − CH_3_]^−^	Methyl gallate ^s^	+	[13]
						124.0175, 78.0136			
20	4.49	C_15_H_18_O_9_		341.0881 (0.8)		179.0352 [M − H − Glc]^−^	Caffeoylglucose	+	[13]
						135.0454 [M − H − Glc − CO_2_]^−^			
21	4.49	C_15_H_14_O_6_	291.0860 (−0.9)		207.0643, 147.0439		Catechin ^s^		[13]
					139.0392, 123.0440				
22	4.55	C_16_H_18_O_9_	355.1023 (−0.1)	353.0878 (3.0)	163.0386 [M + H − H − Glc − OCH_2_]^+^	191.0564 [M − H − Glc]^−^	Chlorogenic acid ^s^	−	[16]
					145.0280, 117.0329				
23	4.77	C_17_H_24_O_9_	373.1487 (−1.6)				Syringoside ^s^	−	[17]
24	4.96	C_9_H_8_O_4_		179.035 4 (2.3)		135.0459 [M − H − CO_2_]^−^	Caffeic acid ^s^	+	[13]
25	5.15	C_11_H_10_O_5_	223.0595 (−2.5)	221.0456 (0)	162.0302 [M + H − H − CH3 − HCOOH]^+^	206.0213 [M − H − CH_3_]^−^	Isofraxidin ^s^	−	[16]
						190.9989 [M − H − OCH_3_]^−^			
						177.0184 [M − H − CO_2_]^−^			
						163.0003 [M − H − OCH_2_ − CO]^−^			
26	5.36	C_20_H_23_O_4_N	342.1695 (−1.3)	340.1557 (0.8)	297.1110 [M + H − HCN − H_2_O]^+^	310.1088 [M − H − OCH_2_]^−^	Magnoline	−	[14]
					282.0881 [M + H − HCN − H_2_O − CH_3_]^+^	252.0404 [M − H − CO_2_ − NH_3_ − HCNN]^−^			
					237.0902, 191.0848, 58.0680	224.0509 [M − H − CO_2_ − NH_3_ − HCNN − H_2_O]^−^			
						162.0180, 118.0288,79.9591			
27	5.51	C_19_H_18_O_11_	423.0925 (0.7)	421.0780 (0.8)	405.0776 [M + H − H_2_O]^+^	331.0459 [M − H − HCOOH − CO_2_]^−^	Mangiferin	−	[18]
					339.0520, 327.0493, 303.0478	271.0253 [M − H − Xyl]^−^			
					285.0417	259.0230 [M − H − Glc]^−^			
					273.0395,				
28	5.54	C_17_H_20_N_4_O_6_	377.1450 (−1.6)		359.1339, 243.0878, 198.0644		Riboflavin	−	[19]
29	5.97	C_17_H_20_O_9_		367.1038 (0.9)		191.0561	3-Feruloyquinic acid	−	[13]
30	6.07	C_23_H_28_O_11_		479.1569 (2)		449.1433, 327.1081	Paeoniflorin ^s^	−	[20]
						255.0647, 165.0568, 121.0297			
31	6.08	C_9_H_10_O_5_		197.0458 (1.4)		169.0147, 124.0175, 78.0178	Syringic acid ^s^	+	
32	6.2	C_9_H_8_O_3_		163.0400 (−0.4)		119.0505 [M − H − CO_2_]^−^	*trans-*p-Coumaric acid ^s^	+	
						93.0352 [M − H − CO − C_2_H_2_O]^−^			
33	6.48	C_34_H_46_O_18_	743.2744 (−1.8)	741.2628 (2.2)		579.2111 [M − H − Glc]^−^	Eleutheroside E ^s^	−	[13]
						417.1579 [M − H − 2Glc]^−^			
34	6.48	C_28_H_36_O_13_		579.2100 (2.9)			Eleutheroside E1	−	[13]
35	6.82	C_10_H_10_O_4_		193.0508 (0.8)		178.0263 [M − H − CH_3_]^−^	Ferulic acid ^s^	−	[13]
						134.0375 [M − H − CH_3_ − CO_2_]^−^			
36	6.9	C_20_H_22_O_8_	391.1372 (−3.9)	389.1252 (2.7)	229.0859 [M + H − H − Glc]^+^	227.0718 [M − H − Glc]^−^	Polydatin ^s^	+	[21]
						185.0612 [M − H − Glc − C_2_H_2_O]^−^			
37	6.91	C_14_H_12_O_3_	229.0850 (3.9)	227.0715 (0.7)	183.0788	185.0601, 143.0508	Resveratrol ^s^	+	[22]
					165.0690 [M + H − H_2_O − HCOOH]^+^				
					107.0489, 91.0540				
38	6.95	C_26_H_28_O_13_		547.1479 (4)			6-C-Arabinosyl-8-C-Glucosyl-Chrysin	+	[23]
						487.1279			
						457.1169 [M − H − C_3_H_6_O − OCH_2_]^−^			
						427.1052			
						409.0946, 367.0839			
						337.0734 [M − H − Glc − OCH_2_]^−^			
39	7.04	C_27_H_30_O_16_	611.1584 (−3.6)	609.1482 (3.8)	465.1016 [M + H − H − Rha]^+^	301.0354 [M − H − Rha − Glc]^−^	Rutin ^s^	−	[18]
					303.0501 [M + H − H − Rha − Glc]^+^	271.0228			
40	7.10	C_21_H_18_O_12_		461.0736 (2.2)		285.0416 [M − H − Glc acid]^−^	Scutellarin ^s^	+	[18]
									
41	7.19	C_21_H_20_O_12_		463.0891 (1.9)		301.0360 [M − H − Glc]^−^	Hyperoside ^s^	+	[24]
						271.0250 [M − H − Glc − OCH_2_]^−^			
						255.0310 [M − H − O − Glc − OCH_2_]^−^			
						178.9998, 151.0041			
42	7.21	C_23_H_24_O_13_	509.1271 (−3.6)	507.1156 (2.3)	347.0750 [M + H − H − Glc]^+^,	345.0632 [M − H − Glc]^−^	Viscidulin III-	−	[25]
					332.0523 [M + H − H − Glc − CH_3_]^+^	330.0383 [M − H − Glc − CH_3_]^−^	Glucopyranoside		
					314.0409	315.0174 [M − H − Glc − 2CH_3_]^−^			
43	7.32	C_26_H_28_O_13_		547.1479 (4.0)		487.1279	6-C-Glucosyl-8-C-Arabinosyl-Chrysin		[23]
						457.1169 [M − H − C_3_H_6_O − OCH_2_]^−^			
						427.1052			
						409.0946, 367.0839			
						337.0734 [M − H − Glc − OCH_2_]^−^			
44	7.41	C_21_H_20_O_13_		479.0831 (0.1)		441.0761, 435.0957	Isomyricitrin	−	[26]
						313.0570, 165.0548			
45	7.59	C_25_H_24_O_12_	517.1326 (−2.8)	515.1199 (0.7)	499.1177 [M + H − H_2_O]^+^	353.0889	Cynarin	−	[13]
					163.0382	191.0562			
						179.0347			
46	7.63	C_33_H_40_O_18_		723.2144 (0.2)			Ligustroflavone ^s^	−	[27]
47	7.63	C_31_H_42_O_17_	687.24992 (0.6)	685.2372 (3.4)		523.1831 [M − H − Glc]^−^	Specnuezhenide	−	[15]
						454.1404, 421.1508			
						299.1137, 223.0603			
48	7.74	C_20_H_18_O_11_	435.0913 (−2)	433.0784 (1.7)	303.0493 [M + H − H−Arab]^+^	301.0294 [M − H − Arab]^−^	Quercetin-3-arabinoside	−	[23]
						271.0257 [M − H − Arab − OCH_2_]^−^			
						255.0314, 178.9989, 151.0036			
									
49	7.93	C_21_H_20_O_9_	417.1169 (−2.6)	415.1044 (2.2)	399.1071 [M + H − H_2_O]^+^	325.0692 [M − H − C_3_H_6_O_3_]^−^	Chrysin-8-C-glucoside	+	[23]
					297.0758 [M + H − H − C_4_H_8_O_4_]^+^	295.0624 [M − H − C_4_H_8_O_4_]^−^			
					279.0639 [M + H − H − C_4_H_8_O_4_−H_2_O]^+^	267.0676 [M − H − C_4_H_8_O_4_ − CO]^−^			
					267.0644 [M + H − H − C_4_H_8_O_4_ − 2H_2_O]^+^	149.0237			
50	7.97	C_21_H_20_O_11_	449.1063 (−3.3)	447.0947 (3.1)	303.0488 [M + H − H − Rha]^+^	301.0379 [M − H − Rha]^−^	Quercetrin ^s^	−	[18]
					287.0549 [M + H − H − O − Rha]^+^	271.0265 [M − H − Rha − OCH_2_]^−^			
					129.0543, 85.0289	255.0315, 178.9983			
					85.0286	151.0041			
51	8.22	C_32_H_38_O_16_	679.2215 (−2.6)		533.1669 [M + H − H − Rha]^+^		hexandraside E	−	[14]
					371.1096 [M + H − H − Rha − Glc]^+^				
					315.0457 [M + H − H − Rha − Glc − C_4_H_8_]^+^				
52	8.52	C_38_H_48_O_20_	825.2794 (−2.1)		663.2238 [M + H − H − Glc]^+^		Diphylloside B	−	[14]
					517.1674 [M + H − H − Rha − Glc]^+^				
					355.1199 [M + H − H − Rha−2Glc]^+^				
					299.0562 [M + H − H − Rha−2Glc − C_4_H_8_]^+^				
53	8.53	C_26_H_28_O_11_	517.1693 (−2.2)		355.1169 [M + H − H − Glc]+		Icarrin C	−	[14]
					299.0524 [M + H − H—Glc − C_4_H_8_]^+^				
54	8.58	C_21_H_18_O_11_	447.0912 (−2.2)	445.07858 (2.1)	271.0595 [M + H − H − O − Glc]^+^	269.0459 [M − H − O − Glc]^−^	Baicalin ^s^	+	[23]
55	8.82	C_38_H_48_O_19_	809.28357 (−3.3)	807.2011 (0.33)	663.2266 [M + H − H − Xyl]^+^		Epimedin B	−	[14]
					517.1687 [M + H − H − Xyl − Rha]^+^				
					355.1182 [M + H − H − Xyl − Rha − Glc]^+^				
					299.0542 [M + H − H − Xyl − Rha − Glc − C_4_H_8_]^+^				
56	8.86	C_22_H_22_O_9_	431.1318 (−4.2)	429.1101 (0.3)	269.0802 [M + H − H − Glc]^+^		Ononin	−	
57	8.87	C_21_H_22_O_9_		417.1191 (0)		255.0678 [M − H − Glc]^−^	Polygonimitin B		[18]
						211.0773 [M − H − Glc − CO_2_]^−^			
58	8.89	C_21_H_20_O_11_	449.1063 (−3.3)	447.0946 (3.1)	303.0488 [M + H − H − Rha]^+^	301.0379 [M − H − Rha]−	Isoquercetrin	−	
					287.0549 [M + H − H − O − Rha]^+^	271.0265 [M − H − Rha − CH_2_]^−^			
					129.0543,85.0289	255.0315,178.9983			
59	8.9	C_32_H_38_O_15_	663.2265 (−2.7)	661.2169 (4.7)	517.1667 [M + H − H − Rha]^+^	515.1907	Epimedoside A	−	
					355.1180 [M + H − H − Rha − Glc]^+^	353.1050			
					299.0559 [M + H − H − Rha − Glc − C_4_H_8_]^+^				
60	9.0	C_17_H_14_O_8_	347.0751 (−3.1)		332.0523, 317.0288, 314.0414		Viscidulin III	−	[23]
					289.0340, 169.0125				
61	9.04	C_39_H_50_O_20_	839.2967 (−0.2)		677.2346 [M + H − H − Glc]^+^		Epimedin A	−	
					531.1881 [M + H − H − Glc − Rha]^+^				
					369.1342 [M + H − H − 2Glc − Rha]^+^				
					313.0691 [M + H − H − 2Glc − Rha − C_4_H_8_]^+^				
62	9.15	C_33_H_40_O_16_	693.2365 (−3.4)		547.1791 [M + H − H − Rha]^+^		anhydroicaritin	−	[14]
					531.1895 [M + H − H − Glc]^+^		-3,7-di-*O*-glucoside		
					385.1284 [M + H − H− Rha − Glc]^+^				
					369.1321 [M + H − H − O − Rha − Glc]^+^				
					313.0687 [M + H − H − O − Rha − Glc − C_4_H_8_]^+^				
63	9.15	C_30_H_32_O_13_		599.1787 (2.8)		581.1699 [M − H − H_2_O]^−^	Benzoyloxypaeoniflorin	−	[20]
						477.1593 [M − H − C_7_H_6_O_2_]^−^			
						449.7983 [M − H − C_7_H_6_O_2_ − CO]^−^			
						431.1372 [M − H − H_2_O − Xyl]^−^			
						281.0676 [M − H − H_2_O − Xyl − C_5_H_10_O_5_]^−^			
64	9.45	C_22_H_22_O_10_	447.1269 (−3.8)	445.1143 (0.6)	285.0757 [M + H − H − Glc]^+^,	430.0906 [M − H − CH_3_]^−^	Wogonin-7-*O*-glucoside	−	[23]
					270.0526 [M + H − H − Glc − CH_3_]^+^	268.0456 [M − H − CH_3_ − Glc]^−^			
65	9.51	C_22_H_20_O_12_	477.1026 (−2.3)	475.0892 (2)	301.0706 [M + H − H − Glucuronic acid]^+^	299.0573 [M − H − Glucuronic acid]^−^	5,4′-Dihydroxy-8-methoxy-flavone-7-*O*-glucuronide	+	[23]
					286.0470 [M + H − H − Glucuronic acid − CH_3_]^+^	284.0324 [M − H − Glucuronic acid − CH_3_]^−^			
66	9.8	C_15_H_10_O_7_		301.0356 (0.9)		273.0396 [M − H − CO]^−^	Quercetin	−	[18]
						245.0407 [M − H − CO − H_2_O]^−^			
						178.9978			
						151.0035, 121.0292			
						107.0144			
67	9.9	C_21_H_18_O_11_	447.0919 (−0.7)		271.0595		Baicalein-6-*O*-glucuronide	+	[23]
68	9.96	C_11_H_12_O_4_		207.0667 (2.1)		179.0345, 135.0469	Ethyl Caffeate ^s^		[23]
									
69	9.99	C_22_H_22_O_11_	461.1088 (−0.4)	459.0949 (3.4)	285.0759, 270.0522	283.0624, 268.0387, 175.0256, 113.0261	Wogonoside ^s^	−	[23]
									
70	10.03	C_22_H_20_O_11_	461.1069 (−2)	459.0949 (3.4)	285.0759 [M + H − H − Glucuronic acid]^+^	283.0624 [M − H − Glucuronic acid]^−^	Oroxyloside ^s^	+	[23]
					270.0522 [M + H − H − Glucuronic acid − CH_3_]^+^	268.0387 [M − H − Glucuronic acid − CH_3_]^−^			
71	10.33	C_39_H_50_O_19_	823.30066 (−1.5)		677.2421 [M + H − H − Rha]^+^		Epmedin C	−	[14]
					531.1840 [M + H − H−2Rha]^+^				
					369.1326 [M + H − H−2Rha − Glc]^+^				
					313.0708 [M + H − H−2Rha − Glc − C_4_H_8_]^+^				
72	10.34	C_27_H_30_O_11_	531.1846 (−2.8)		369.1326 [M + H − H − Glc]^+^		Icariside I ^s^	−	[14]
					313.0695 [M + H − H − Glc − C_4_H_8_]^+^				
73	10.55	C_33_H_40_O_15_	677.24256 (−2.1)	675.2326 (4.7)	531.1847 [M + H − H − Rha]^+^	367.1099	Icariin ^s^	+	[14]
					369.1332 [M + H − H − Rha − Glc]^+^	269.0461 [M − H − Rha − Glc]^−^			
					313.0712 [M + H − H − Rha − Glc − C_4_H_8_]^+^				
74	11.3	C_9_H_10_O_3_		165.0557 (0)		150.0333 [M − H − CH_3_]^−^	Paeonol ^s^	+	[28]
						135.0094 [M − H − OCH_2_]^−^			
					122.0385, 91.0208				
75	11.44	C_15_H_10_O_6_		285.0407 (0.8)		241.0517 [M − H − CO_2_]^−^	Kaempferol ^s^	+	[18]
						211.0408 [M − H − CO_2_−OCH_2_]^−^			
						195.0463,167.0509			
76	11.56	C_16_H_12_O_6_		299.0563 (0.7)		284.0327 [M − H − CH_3_]^−^	5,7,4’-Trihydroxy-8-	−	[23]
						240.0424 [M − H − CH_3_ − CO_2_]^−^	methoxyflavone		
						171.0452,153.9909			
						125.9968			
77	11.59	C_16_H_12_O_7_		315.0510 (0)		300.0279 [M − H − CH_3_]^−^	Isorhamnetin ^s^	+	[29]
						282.0178 [M − H − CH3 − H_2_O]^−^			
						255.0240, 151.0016			
78	11.71	C_32_H_38_O_15_	663.3011 (3.1)	661.2163 (−3.5)	517.1667 [M + H − HCOOH]^+^	353.0978 [M − H − Rha − Glc]^−^	Ikarisoside B	+	[26]
					355.1165 [M + H − H − Rha − Glc]^+^				
					299.0599 [M + H − H − Rha − Glc − C_4_H_8_]^+^				
79	11.73	C_15_H_10_O_5_	271.0597 (−1.6)	269.0456(1.9)	253.0500 [M + H − H_2_O]^+^	251.036 [M − H − H_2_O]^−^	Baicalein ^s^	+	[23]
					123.0080	223.0407, 169.0664			
									
80	12.15	C_31_H_36_O_14_		631.2050 (2.8)		481.1616 [M − H − Xyl]^−^	Ikarisoside F	+	[26]
						353.1076, 352.0928, 341.0528			
81	12.37	C_16_H_12_O_4_	269.0805 (−1.2)	267.0664 (0.4)	254.0576, 237.0532	252.0430, 223.0396, 195.0445	Formononetin ^s^	−	[10]
					197.0599	132.0221			
					181.0644, 118.0416				
82	12.65	C_26_H_28_O_10_	501.1744 (−2.3)	499.1618 (1.6)	355.1169 [M + H − H − Rha]^+^	353.1043 [M − H − Rha]^−^	Baohuoside II ^s^	+	[14]
					299.0559 [M + H − H − Rha − C_4_H_8_]^+^	309.0445 [M − H − Rha − CO_2_]^−^			
					147.0645,129.0533				
83	13.34	C_33_H_40_O_15_	677.2417 (−3.4)	675.2326 (4.7)	531.1847 [M + H − H − Rha]^+^	367.1099 [M − H − Rha − Glc]^−^	Baohuoside VII	+	[26]
					369.1324 [M + H − H − Rha − Glc]^+^	352.0997			
					313.0712 [M + H − H − Rha − Glc − C_4_H_8_]^+^				
84	13.49	C_43_H_70_O_15_	827.4768 (−2.3)		455.3660, 437.335		Astragaloside II	−	[10]
					175.0610,157.0474,143.1087				
85	13.74	C_42_H_68_O_13_	781.4736 (0.4)		745.4447		Saikosaponin a	−	[30]
					619.4203, 583.3921				
					455.3486, 437.3374				
					419.3231				
86	13.79	C_16_H_12_O_5_	285.0754 (−1.3)	283.0615 (0.9)	270.0514 [M + H − H − CH_3_]^+^	268.0380 [M − H − CH_3_]^−^	Wogonin ^s^	+	[23]
					179.0488	163.0036			
									
87	13.80	C_32_H_38_O_14_	647.2323 (−1.7)		501.1726 [M + H − H − Rha]^+^		Sagittatoside B	+	[26]
					465.1557 [M + H − H − Rha − HCOOH]^+^				
					409.0917, 355.1171				
					299.0594				
88	13.81	C_16_H_12_O_5_	285.0754 (−1.3)	283.0615 (0.9)	270.0514 [M + H − H − CH_3_]^+^	268.0380 [M − H − CH_3_]^−^	Oroxylin A ^s^	+	[23]
					179.0488	163.0036			
89	13.82	C_32_H_38_O_14_	647.2323 (−1.7)		629.2323, 501.1726,		2′′-*O*–Rhamnosyl-ikarisoside A		[26]
					465.1557, 355.1171, 299.0059				
90	13.9	C_33_H_40_O_14_	661.2470 (−3.1)	659.2374 (4.4)	515.1897 [M + H − H − C_6_H_10_O_4_]^+^	366.1127	2′′-*O*-Rhamnosyl-ikariside II	+	[14]
					369.1333, 355.0809, 313.0703				
91	13.96	C_15_H_10_O_4_	255.0642 (−3.9)	253.0708 (0.7)	153.0177 [M + H − H − C_5_H_8_O − H_2_O]^+^	209.0609 [M − H − CO_2_]^−^	Chrysin ^s^	+	[23]
					103.0544	143.0505, 63.0276			
92	14.01	C_17_H_14_O_6_	315.0856 (−2.3)	313.0718 (0.2)	300.0618, 285.0396	298.0485, 283.0252, 211.0396	Kumatakenin		[29]
					257.0440, 182.9919, 154.9964	155.0506			
93	14.5	C_24_H_32_O_7_	433.2215 (−1.3)		415.2106 [M + H − H_2_O]^+^		Schisandrin ^s^	+	[31]
					400.1869 [M + H − H_2_O − CH_3_]^+^				
					384.1923 [M + H − H − OCH3 − H_2_O]^+^				
					359.14907 [M + H − H_2_O − C_4_H_8_]^+^				
					315.1223				
94	14.63	C_27_H_30_O_10_	515.1901 (−2)	513.1780 (2.7)	369.1321 [M + H − H − Rha]^+^	367.1134 [M − H − Rha]^−^	Icarisid II ^s^	+	[14]
					313.0700 [M + H − H − Rha − C_4_H_8_]^+^	351.0882 [M − H − O − Rha]^−^, 323.0912			
95	15.2	C_45_H_72_O_16_	869.4881 (−1.4)		689.4234, 437.3396		Astragaloside I ^s^	−	[10]
					217.0720, 157.0506, 143.1059				
96	15.5	C_23_H_28_O_7_	417.1890 (−4.2)		399.1815 [M + H − H_2_O]^+^		Schisandrol B	−	[31]
					384.1501 [M + H − H_2_O − CH_3_]^+^				
					368.1618 [M + H − H_2_O−OCH_3_]^+^				
					357.1394 [M + H − H_2_O − C_3_H_6_]^+^				
					343.1187 [M + H − H_2_O − C_4_H_8_]^+^				
97	16.02	C_30_H_48_O_5_	489.3566 (−1.8)	487.3434 (1)	453.3324 [M + H − H − 2H_2_O]^+^	469.3361 [M − H − H_2_O]^−^	Tormentic acid	+	[15]
					407.3254 [M + H − HCOOH−2H_2_O]^+^	407.1533			
					201.1702, 127.0767				
98	16.07	C_21_H_20_O_6_	369.1323 (−2.6)		313.0693		Icaritin	−	[14]
99	16.08	C_15_H_10_O_5_	271.0596 (−2)	269.0460 (1.5)		225.0560, 241.0514, 197.0608, 182.0375	Emodin ^s^	+	[18]
100	17.88	C_23_H_30_O_6_	403.2112 (−0.8)		388.1848 [M + H − H − CH_3_]^+^,		Schisanhenol	−	[31]
					372.1919 [M + H − H − OCH_3_]^+^				
					371.1859 [M + H − H − CH_3_OH]^+^				
					333.1326, 302.1149				
101	19.09	C_28_H_34_O_9_	515.2264 (−2.3)		415.2009 [M + H − H − C_4_H_6_COOH]^+^		Schisantherin B	−	[31]
					385.1637 [M + H − H − C_4_H_6_COOH − OCH_2_]^+^				
					355.1534, 343.1160, 316.0939				
102	20.31	C_24_H_32_O_6_	417.2259 (−3)		402.1999 [M + H − H − CH_3_]^+^		Schizandrin A ^s^	−	[31]
					386.2059 [M + H − H − OCH_3_]^+^				
					347.1480 [M + H − H − C_5_H_10_]^+^				
					316.1289 [M + H − H − C_5_H_10_ − OCH_3_]^+^				
					301.1058, 285.1104, 273.1091, 242.0911				
103	21.3	C_23_H_28_O_6_	401.1948 (−2.6)		386.1728 [M + H − H − CH_3_]^+^		γ-Schizandrin B ^s^	−	[31]
					331.1168 [M + H − H − C_5_H_10_]^+^				
					300.0987, 242.0929				
104	21.87	C_22_H_24_O_6_	385.1638 (−1.9)		355.1561 [M + H − H − OCH_2_]^+^		Schizandrin C	−	[31]
					315.0895 [M + H − H − C_5_H_10_]^+^				
					285.0757 [M + H − H − C_5_H_10_−OCH_2_]^+^				
					257.0809, 242.0588, 227.0703, 153.0663				
105	22.98	C_32_H_50_O_5_	515.3723 (−1.6)	513.3594 (1.7)	409.3466, 191.1793	495.3496 [M − H − H_2_O]^−^	19α−Hydroxyl−3−acetyl ursolic acid	−	[15]
						469.3720 [M − H − CO_2_]^−^			
106	23.56	C_30_H_48_O_3_		455.3537 (1.3)			Oleanic acid ^s^	−	[15]
107	23.73	C_30_H_48_O_3_		455.3537 (1.3)			Ursolic acid	−	[15]

^“s”^ Identified with reference compounds; “+”: detected and “−”not detected.

**Table 3:** Contents of baicalein, paeonol, wogonin and emodin in HGKYC (μg/g, *n* = 3, mean ± SD).

Batch	Baicalein	Paeonol	Wogonin	Emodin
1	1893 ± 32	160 ± 2.2	542 ± 6.7	590 ± 7.2
2	17557.2	14557.2	48357.2	55757.2
3	19247.2	17547.2	56747.2	60247.2
